# Mechanism of the entire overdischarge process and overdischarge-induced internal short circuit in lithium-ion batteries

**DOI:** 10.1038/srep30248

**Published:** 2016-07-22

**Authors:** Rui Guo, Languang Lu, Minggao Ouyang, Xuning Feng

**Affiliations:** 1 State Key Laboratory of Automotive Safety and Energy, Tsinghua University, Beijing 100084, China

## Abstract

Lithium-ion batteries connected in series are prone to be overdischarged. Overdischarge results in various side effects, such as capacity degradation and internal short circuit (ISCr). However, most of previous research on the overdischarge of a cell was terminated when the cell voltage dropped to 0 V, leaving the further impacts of overdischarge unclear. This paper investigates the entire overdischarge process of large-format lithium-ion batteries by discharging the cell to −100% state of charge (SOC). A significant voltage platform is observed at approximately −12% SOC, and ISCr is detected after the cell is overdischarged when passing the platform. The scanning electron microscopy (SEM) and X-ray diffraction (XRD) results indicate that the overdischarge-induced ISCr is caused by Cu deposition on electrodes, suggesting possible Cu collector dissolution at the voltage platform near −12% SOC. A prognostic/mechanistic model considering ISCr is used to evaluate the resistance of ISCr (*R*_*ISCr*_), the value of which decreases sharply at the beginning of ISCr formation. Inducing the ISCr by overdischarge is effective and well controlled without any mechanical deformation or the use of a foreign substance.

Lithium-ion batteries are currently used as power sources for electronic devices due to their high energy density and extended lifespan among comparable battery technologies^1^. However, the safety of lithium-ion batteries must be guaranteed before widespread application^2^,^3^,^4^,^5^.

The safety of lithium-ion batteries exposed to extreme conditions has been analyzed in previous studies in terms of thermal runaway^6^,^7^, overcharge^8^, overdischarge^9^,^10^, and internal short circuit (ISCr)^11^,^12^,^13^. Overdischarge is a common type of abuse that may lead to safety problems, such as ISCr^9^. Batteries are increasingly subjected to the conditions of overdischarge as greater numbers of cells are connected in series for a system requiring high voltage, such as electric vehicles^14^. Therefore, overdischarge and its impact on batteries must be investigated.

Several previous studies have cast light on the overdischarge mechanisms of lithium-ion batteries^9^,^15^,^16^,^17^. The anode potential increases abnormally during overdischarge; thus, the Cu current collector of the cell is oxidized to Cu^2+^ 9,14. Simultaneously, over-deintercalation of lithium at the anode during overdischarge causes decomposition of the solid electrolyte interface (SEI), and the decomposition of SEI generates gases, including carbon dioxide^15^. New SEI films form on the anode when the cell is recharged. The growth of the SEI films can lead to degradation of the electrochemical charge transfer processes in the electrodes^17^, as indicated by the impedance increment at low frequency^9^,^18^. Moreover, the morphology of the cathode materials also changes during overdischarge. The side reactions that occur during extreme overdischarge result in the solid-state amorphization of the transition metal compounds^16^. The changes in the morphology of the components within the lithium ion battery lead to capacity degradation. The dissolution of the Cu collector also affects the lifespan of the battery^17^.

Researchers have proposed several approaches to diminish the consequences of overdischarge. Three-electrode measurement was introduced for overdischarge testing to monitor the over-potential at the anode. Lee *et al*. avoided the high-level potential at the anode by adopting an Li_2_NiO_2_-added cell, which exhibits a near-cathode-limiting configuration^14^. Moreover, Kim *et al*. used succinonitrile as an electrolyte additive, which can form a passive layer on the Cu collector, to avoid Cu dissolution during overdischarge^19^.

However, most tests in the literature were terminated when the cell voltage dropped to 0 V, leaving the further influences of overdischarge unclear. Cells are connected in series in actual battery packs, and a cell voltage lower than 0 V can occur when a cell is overdischarged. Overdischarge can lead to ISCr, which may cause thermal runaway during cycling. For example, Maleki *et al*. reported incidental ISCr after the cell was overdischarged and cycled^9^. Brand *et al*. studied the process of overdischarge and inferred the occurrence of ISCr from the temperature^10^. The possible hazards of ISCr remain unknown due to the insufficient number of studies to reveal the entire overdischarge process.

ISCr in lithium-ion batteries is under intensive study because of its significant impact on the batteries’ safety. Several approaches have been proposed to induce or simulate ISCr; however, most include mechanical deformation or the introduction of a foreign substance. Maleki *et al*.^20^, Cai *et al*.^21^ and Zhang *et al*.^22^,^23^ created ISCr by mechanical deformation and structural changes. Orendorff *et al*.^24^, Santhanagopalan *et al*.^25^ and Stringfellow *et al*.^26^ induced ISCr by introducing a foreign substance into the lithium-ion battery. The current study found that ISCr can be induced through deep overdischarge without mechanical destruction or foreign substances.

This work studies the mechanisms of forced overdischarge for large-format lithium-ion batteries. The overdischarge process is divided into three stages according to the characteristics of the entire voltage curves during overdischarge. The impact of overdischarge on the lithium-ion batteries at different stages is analyzed. Severe overdischarge, i.e., SOC < −12%, results in variant levels of ISCr caused by Cu foil dissolution and deposition. The resistance of ISCr (*R*_*ISCr*_) quantitatively reflects the level of ISCr; therefore, *R*_*ISCr*_ is analyzed by recharging the battery after the overdischarge tests.

*R*_*ISCr*_ is evaluated using a prognostic/mechanistic model^8^,^27^,^28^ combined with ISCr^29^ by fitting the observed open circuit voltage (OCV) data during self-discharge. Furthermore, SEM and XRD experiments are conducted to study the surface morphology and characterization of the materials on electrodes. The results supported the notion that Cu deposition occurs during the overdischarge of lithium-ion batteries, which in turn causes ISCr.

## Results

### Voltage curve during overdischarge

**Figure 1:**
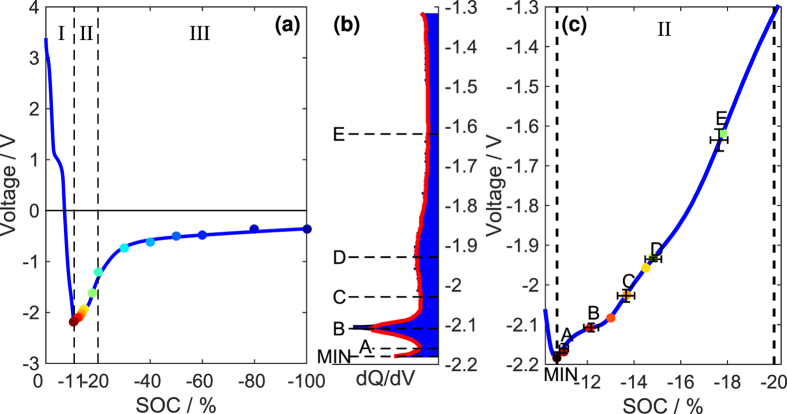
Voltage analysis during overdischarge. (**a**) voltage profile during overdischarge and terminal conditions of cells 2–16 dotted in the descending order of SOC, (**b**) incremental capacity analysis of Stage II with peaks and valleys marked, (**c**) enlarged view of Stage II showing the inflection points with error bars.

The voltage during overdischarge is shown in Fig. 1(a). The overdischarge profile can be approximately divided into 3 stages according to the characteristics of the voltage variations. In Stage I (−11% < SOC ≤ 0%), the voltage dropped rapidly from 3.4 V to the minimum voltage of approximately −2.19 V, following a clear platform at approximately 1 V. In Stage II (−20% < SOC ≤ −11%), the voltage stopped declining and rebounded gradually with several fluctuations. In Stage III (−100% < SOC ≤ −20%), the voltage underwent a monotonic gradual increase asymptotically to 0 V without fluctuations.

The voltage drop in Stage I is caused by the increasing potential of anode and the decreasing potential of cathode; because overdischarge leads to deintercalation of Li^+^ from the anode, and insertion into the cathode. In Stage II, when the anode potential reaches approximately 3.4~3.5 V^15^,^19^, anodic corrosion of the Cu collector is triggered; the anode potential thus enters an electrochemical reaction platform for the Cu dissolution. Copper ions dissolved in the electrolyte can travel through the separator and deposit on the cathode; the cathode potential thus increases due to the reduction of copper ions. The overpotential for Cu dissolution can account for the voltage valley at approximately −11% SOC. In Stage III, the electrochemical reactions of Cu dissolution and deposition continue, and the internal short becomes more severe, with a decrease of *R*_*ISCr*_. Therefore, the absolute value of the voltage, which is the product of the overdischarge current and *R*_*ISCr*_, decreases and approaches zero.

**Figure 2:**
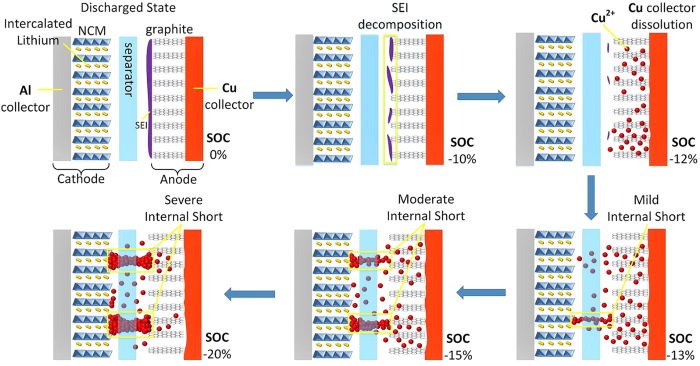
Copper dissolution and deposition during overdischarge and the formation of internal short circuit.

The cells under overdischarge that were terminated in Stage I exhibited no discernible changes in properties, whereas in Stage III, the voltage increased slowly asymptotically to 0 V, suggesting the occurrence of severe ISCr. However, in Stage II, the properties of the voltage curve were more complex because the results varied at different terminal conditions. To detail the variations in voltage and its influence on overdischarge, the voltage curve of stage II was analyzed by incremental capacity analysis^30^, as shown in Fig. 1(b); the peaks and valleys of the incremental capacity curve indicate the inflection points of the voltage curve, denoted as MIN, A, B, C, D and E in Fig. 1(c). The terminal conditions in Stage II were chosen to be near the inflection points according to the voltage curve analysis above. Inflection point B in Fig. 1(b) is located at a significant peak of incremental capacity, representing the electrochemical reaction platform where Cu collector dissolution is inferred. Figure 2 illustrates the process of Cu dissolution during overdischarge and the formation of the ISCr induced by overdischarge. The internal short caused by Cu deposition occurs after the cell is overdischarged to SOC < −12% and becomes more severe during the overdischarge process.

### Recharging after different degrees of overdischarge

Cells were recharged with 8.33 A (1/3C) current after the overdischarge tests terminated under different conditions (see Supplementary Table S1). The recharge experiments were separated into two categories (with and without ISCr) according to the occurrence of ISCr.

Cells 2, 3, and 4 did not exhibit ISCr after being overdischarged to MIN, A and B (Fig. 1(c)), respectively, as they could be fully recharged and cycled without any signs of ISCr or significant capacity loss. The results of the non-ISCr cells suggest that if the overdischarge is terminated before point B at approximately −12% SOC (the first platform after the occurrence of the minimum voltage), the cell can be fully charged back and reused with only minor side effects.

**Figure 3:**
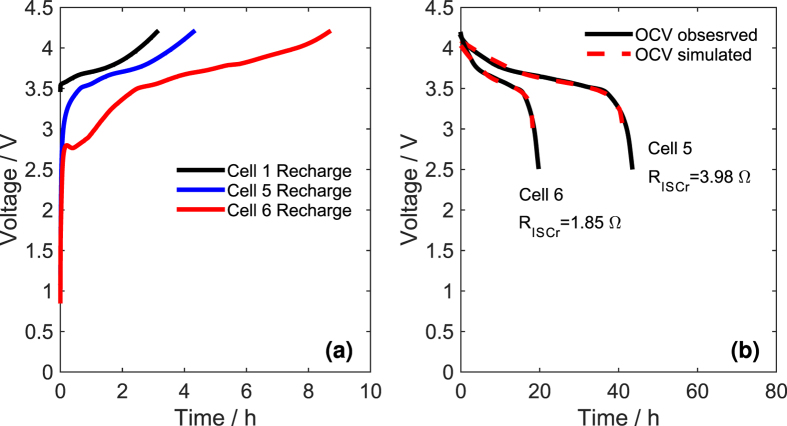
The recharge process and depleting OCV. (**a**) recharge process of cells 5 and 6 with ISCr, compared with normal cell 1, (**b**) the depleting OCV and simulation results of cells 5 and 6.

The other samples (with ISCr) overdischarged over point B showed evident characteristics of ISCr with different resistances (*R*_*ISCr*_). Cells 5 and 6, overdischarged to −13.0% and −13.7% respectively, could be fully recharged with 8.33 A (1/3C) current (Fig. 3(a)). After the recharge, cells 5 and 6 displayed significant self-discharge, and the depleting OCV of cells 5 and 6 is shown in Fig. 3(b). It was more difficult to fully charge cell 6 compared to cell 5, as the charging time was longer for cell 6. Moreover, the OCV of cell 6 depletes more rapidly than that of cell 5. This phenomenon suggests that cells 5 and 6 both suffered from ISCr, and the *R*_*ISCr*_ of cell 6 was lower than that of cell 5.

Cells that had been overdischarged to SOC < −14.5% could not be fully recharged to 4.2 V with 8.33 A (1/3C) current. During the recharge process, their voltages increased once the recharge began but soon reached a stable value. The stable voltage during the recharge process becomes lower as the overdischarge increased further, suggesting a lower *R*_*ISCr*_.

### Estimation of *R*_*ISCr*_ using a prognostic/mechanistic model

**Figure 4:**
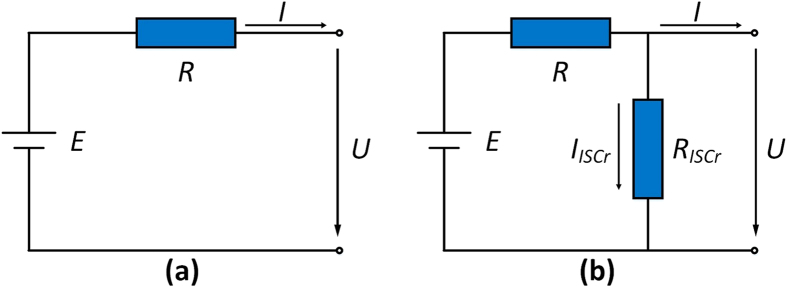
Equivalent circuit model. (**a**) a normal battery, (**b**) a battery with ISCr.

*R*_*ISCr*_ can be quantitatively evaluated by analyzing the depleting OCV after the cell is fully recharged. The *R*_*ISCr*_ of cells 5 and 6 are estimated using prognostic/mechanistic model^8^,^27^,^28^ combined with an equivalent circuit model of a battery with ISCr^29^, as shown in Fig. 4(b). According to the equivalent circuit model, the simulated OCV, denoted as *V*_sim_(*t*) in Eqn. (1), is equal to the voltage caused by *R*_*ISCr*_.


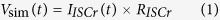


The current *I*(*t*) is a constant 0 because the voltage of the battery is observed at an open circuit, as shown in Eqn. (2). Combining Eqn. (2) with Kirchhoff’s current law in Eqn. (3), we obtain Eqn. (4), which indicates that the current through *R*_*ISCr*_ and internal resistance *R* should be the equivalent at any given time.


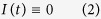



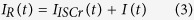



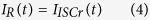


Eqn. (5) is written according to Kirchhoff’s voltage law, where *E*(*t*) denotes the electromotive force given by the prognostic/mechanistic model in Eqn. (6). *V*_*p*_(*y*(*t*)) and *V*_*n*_(*x*(*t*)) denote the cathode potential and anode potential, respectively. *y*(*t*) is defined as a customized variable in the half-cell tests instead of the stoichiometric lithium content in the cathode^8^. Furthermore, *x*(*t*) refers to the value *x* in Li_x_C_6_^31^. Half-cells with Li_y_Ni_1/3_Co_1/3_Mn_1/3_O_2_(NCM)/Li and graphite/Li were made and cycled at C/20 current at 25 °C to acquire *V*_*p*_(*y*) and *V*_*n*_(*x*) (see Supplementary Fig. S1).


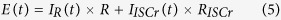



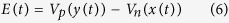


Eqn. (7), which is derived from Eqns (4, 5, 6), determines the current during self-discharge. By combining Eqn. (7) with Eqn. (1), we obtain the expression of *V*_sim_(*t*) in Eqn. (8).


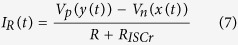



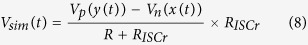


The cathode potential *V*_*p*_(*y*(*t*)) and anode potential *V*_*n*_(*x*(*t*)) change over time during the self-discharge process because *y*(*t*) and *x*(*t*) are functions of time, as shown in Eqns (9, 10), where *y*_0_ (*x*_0_) denotes the initial value of *y* (*x*), *C*_*p*_ (*C*_*n*_) represents the capacity of the cathode (anode), and 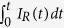 is the integral of the self-discharge current. The cathode potential and anode potential during self-discharge can be determined by combining *y*(*t*) and *x*(*t*) with the separated half-cell quasi-equilibrium voltage curve, as shown in Supplementary Fig. S1.


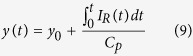



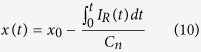


Therefore, the OCV during self-discharge can be simulated from Eqns (7, 8, 9, 10) by choosing appropriate settings for [*R*, *R*_*ISCr*_, *y*_0_, *x*_0_, *C*_*p*_, *C*_*n*_] using an optimization method, such as a genetic algorithm, as in refs 8,28. The root mean squared error (RMSE) between the simulated open circuit voltage *V*_sim_(*t*) and the observed open circuit voltage is calculated to evaluate the degree of coincidence, as shown in Eqn. (11). n is the length of data used for the simulation, and 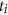 represents the time points.


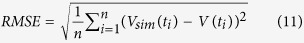


Figure 3(b) compares the simulated OCV with the observed OCV for cells 5 and 6. The simulated voltage curves fit the experimental observations well, indicating that the model and identified parameters approximately reflect the ISCr of the overdischarged cells.

The 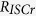 of the cells that could not be fully recharged with 8.33 A (1/3C) current is estimated simply dividing the stable voltage by the charging current, because all of the charging current is entirely bypassed by *R*_*ISCr*_.

**Figure 5:**
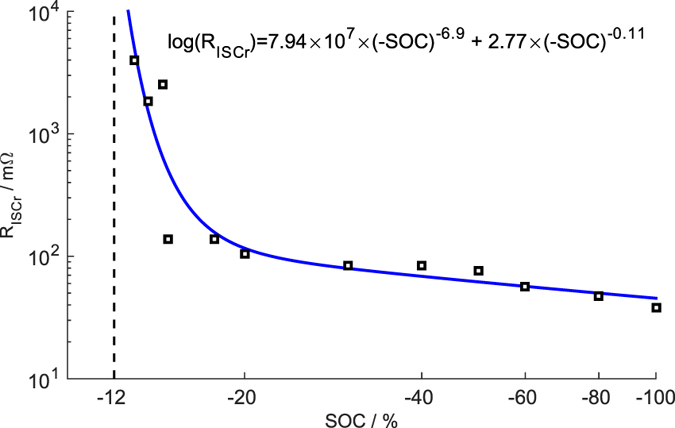
The relationship between *R*_*ISCr*_ and terminal SOC.

Figure 5 shows the relationship between the estimated *R*_*ISCr*_ and the overdischarge SOC. The results suggest that ISCr occurs after the inflection point B at approximately −12% SOC, where the first platform after the minimum voltage is located. The *R*_*ISCr*_ declines with a lower overdischarge SOC. This method of inducing ISCr by overdischarge is effective and can be well controlled.

### SEM and XRD results

**Figure 6:**
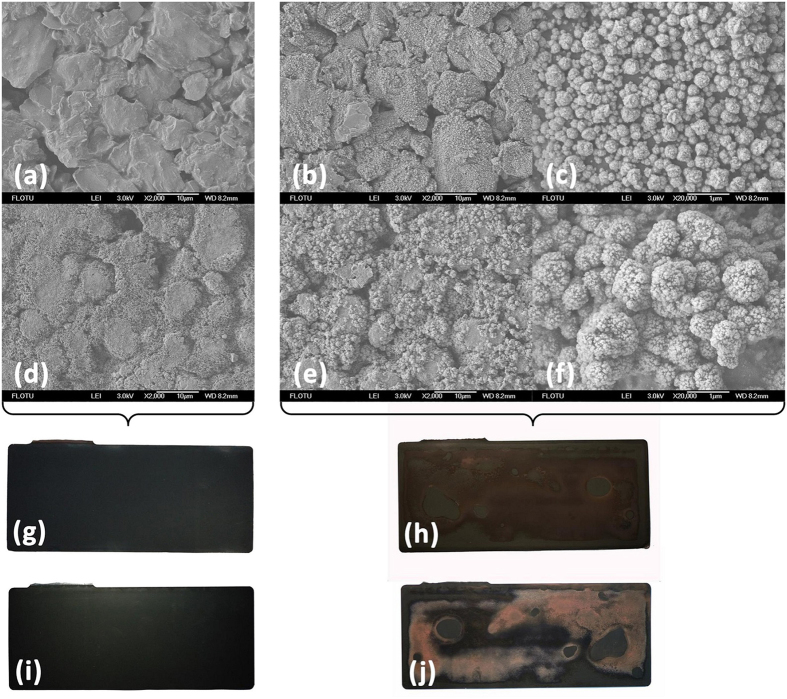
SEM images and digital photographs of cells 1 (SOC = 0%) and 10 (SOC = −20%). (**a**) anode of cell 1 (SEM image), (**b**) anode of cell 10 (SEM image), (**c**) anode of cell 10 under high-magnification (SEM image), (**d**) cathode of cell 1 (SEM image), (**e**) cathode of cell 10 (SEM image), (**f**) cathode of cell 10 under high-magnification (SEM image), (**g**) anode of cell 1 (digital photograph), (**h**) anode of cell 10 (digital photograph), (**i**) cathode of cell 1 (digital photograph), (**j**) cathode of cell 10 (digital photograph).

The SEM and XRD results reveal the surface morphology and structural characterization of ISCr induced by overdischarge. Digital photographs of the electrodes dissembled from cells 1 and 10, which were dismantled after the overdischarge test, are shown in Fig. 6(g–j). Both the cathode and anode of cell 10 are stained with Cu deposition, which is irregularly observed throughout the entire electrodes.

The SEM morphologies of the materials on the electrodes from cells 1 and 10 are compared in Fig. 6(a–f). Figure 6(a,d) show normal graphite and Li_y_Ni_1/3_Co_1/3_Mn_1/3_O_2_ materials on the anode and cathode, respectively. The surface of the graphite anode of cell 1 is smooth, and the cathode is flat with a porous structure. However, after overdischarge to −20% SOC (as in cell 10), the smooth graphite anode is dotted with small spherical depositions, as shown in Fig. 6(b,c), whereas the cathode of cell 10 is also contaminated by larger spherical depositions on the cathode materials, as shown in Fig. 6(e,f).

**Figure 7:**
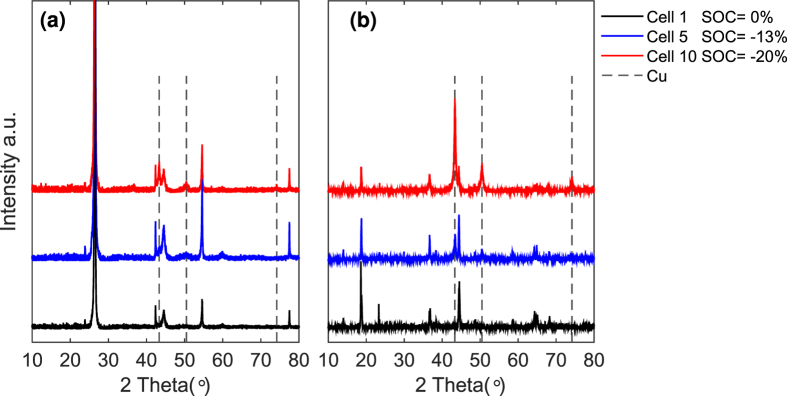
XRD results of cells 1, 5 and 10. (**a**) anode (graphite), (**b**) cathode (NCM).

The XRD results in Fig. 7 suggest that Cu deposition increases gradually on both the anode and cathode during the entire overdischarge process. The emerging peaks of Cu support the previous assumption that Cu foil dissolution and deposition occur on the electrodes during overdischarge.

The SEM and XRD results, in combination with the previous analysis of ISCr induced by overdischarge, demonstrate that the Cu foil dissolution is caused by overdischarge and that the subsequent deposition of cupric ion on the electrodes results in ISCr when Cu deposition penetrates the separator and bridges the two electrodes. As the duration of overdischarge increases, Cu becomes further dissolved and deposited between the electrodes, resulting in more severe ISCr with a lower *R*_*ISCr*_.

## Discussion

This research investigates the entire overdischarge process and overdischarge-induced ISCr of large-format Li-ion batteries with an Li_y_Ni_1/3_Co_1/3_Mn_1/3_O_2_ (NCM) cathode and graphite anode. The voltage of the overdischarged battery decreases to the minimum value at approximately −11% SOC and then increases with several inflection points, eventually asymptotically approaching 0 V. A significant peak of incremental capacity which represents the electrochemical reaction platform of Cu collector dissolution is observed at approximately −12% SOC.

Neither ISCr nor capacity fading occurs if the overdischarge is terminated before −12% SOC, where the first platform after the minimum voltage is located. However, if the terminal SOC is lower than −12%, then the battery suffers from ISCr, which is the result of Cu foil dissolution and deposition on electrodes according to the SEM and XRD results.

The *R*_*ISCr*_ induced by overdischarge is evaluated using an equivalent circuit model with ISCr and a prognostic/mechanistic model. *R*_*ISCr*_ sharply decreases, from −12% to −15% SOC, at the beginning of internal short formation, after which *R*_*ISCr*_ continues to decrease, albeit more gradually.

The overdischarge-induced ISCr is likely to occur when lithium-ion batteries are connected in series with great inconsistency. Moreover, the ISCr induced by overdischarge is well controlled without any mechanical deformation or foreign substance.

The authors wish to note that the capacity ratios of negative to positive electrode have impact on the electrochemical reaction of Cu oxidation. To be specific, given an increased capacity ratio of negative and positive electrode, i.e. more active materials on the negative electrode, the occurrence of the potential of Cu dissolution at the negative electrode will be delayed, thereby postponing the Cu collector dissolution to an SOC more negative than −12% (−15%, −20% etc.).

## Methods

### Performance test

A 25 Ah commercial pouch Li-ion cell with a hard steel case was tested in this study. The cathode material of the battery was NCM, and the anode was graphite; the capacity ratio of negative to positive electrode is 1.18. The performance tests were conducted before the overdischarge tests. The battery was cycled between the voltage limits of 4.2 V and 2.75 V with C/3 current at 25 °C, as listed in Supplementary Table S2. The discharge capacity measured in Step 7 is regarded as the original discharge capacity of the battery.

### Overdischarge test

**Figure 8:**
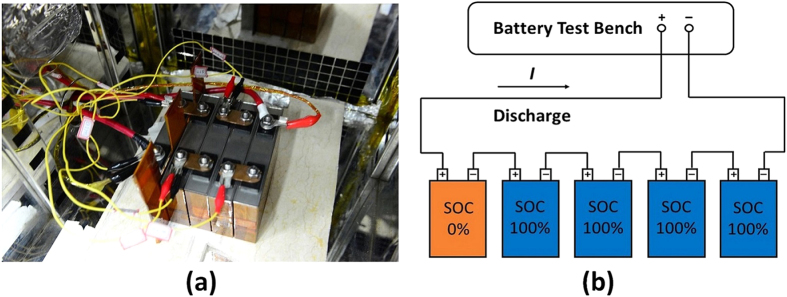
The experiment setup of the overdischarge test. (**a**) Digital photograph of the batteries for the overdischarge test, (**b**) circuit connection diagram of the overdischarge test.

The overdischarge tests were performed in an explosion-proof chamber using a battery test bench manufactured by Digatron^®^ (Digatron Power Electronics GmbH, Tempelhofer Str. 12–14, 52068 Aachen, Germany). In the test, a fully discharged battery was connected in series with 4 auxiliary batteries, and the voltages were monitored. Figure 8 illustrates how the batteries were connected and placed in the overdischarge tests. Supplementary Table S1 summarizes the experimental settings of the 16 batteries. The overdischarge tests covered a wide range of terminal SOCs. Experiments 12–16 were performed to investigate the overall voltage variation of the battery during overdischarge, and a typical voltage curve was drawn (Fig. 1(a)). In experiments 2–9, the terminal conditions were chosen according to the analysis of the overall voltage variation during overdischarge (Fig. 1(c)).

### Recharge test

The recharge tests were conducted on the battery after the overdischarge tests to analyze the impact of overdischarge and estimate the *R*_*ISCr*_ induced by overdischarge. After being overdischarged once, cells were recharged with 8.33 A (1/3C) current and cycled, if possible. If the cell could be fully recharged and exhibited stable static OCV without self-depleting, then the cell was cycled with the same profile as the performance test listed in Supplementary Table S2 to analyze the capacity degradation. If the cell was successfully recharged to 4.2 V but exhibited a significant decrease in OCV, then the cell was considered to have ISCr, and its OCV was further investigated. If the cell could not be charged to 4.2 V, then the recharging was held until its voltage reached a stable value.

### SEM test

Cells were dismantled after the overdischarge test, and the surface morphology of the electrodes was analyzed by an SEM manufactured by JEOL^®^ (3-1-2 Musashino, Akishima, Tokyo 196-8558, JAPAN). The tested cells included cells 1 and 10. Cell 1 was not overdischarged as a control experiment, and cell 10 was overdischarged to −20% SOC.

### XRD test

Cells 1, 5, and 10 were studied using an XRD manufactured by Bruker^®^ (Bruker AXS GmbH, Östliche Rheinbrückenstr. 49, 76187 Karlsruhe, Germany) to analyze the structural characterization of the active materials on both electrodes.

### Half-cell test

The electrochemical properties of electrodes were investigated by half-cell tests. Half-cells with NCM/Li and graphite/Li were made from pieces cut from the electrodes of the battery used in this work. The half-cells were cycled at C/20 current at 25 °C to simulate quasi-equilibrium electrochemical conditions. No significant differences in capacity and electrochemical property were observed between the electrode materials of cell 1 (not overdischarged) and cell 3 (overdischarged to −11%). The OCV of the cathode/anode (Supplementary Fig. S1) is defined as the average of low current charge and discharge curves.

## Additional Information

**How to cite this article**: Guo, R. *et al*. Mechanism of the entire overdischarge process and overdischarge-induced internal short circuit in lithium-ion batteries. *Sci. Rep.* **6**, 30248; doi: 10.1038/srep30248 (2016).

## Supplementary Material

Supplementary Information
