# Supplementary material for: Mechanism of the entire overdischarge process and overdischarge-induced internal short circuit in lithium-ion batteries
Source: Sci Rep. 2016 Jul 22;6:30248. doi: 10.1038/srep30248 (PMC4957210; doi:10.1038/srep30248)
Supplement: Supplementary Information [file srep30248-s1.pdf]

Supplementary Information

Mechanism of the entire overdischarge process and overdischarge-induced internal short circuit in lithium-ion batteries

Rui Guo, Languang Lu, Minggao Ouyang*, Xuning Feng

State Key Laboratory of Automotive Safety and Energy,
Tsinghua University, Beijing 100084, China

*ouymg@tsinghua.edu.cn

Supplementary Figure S1. The open circuit voltage (OCV) of the cathode/anode acquired from the half-cell test.

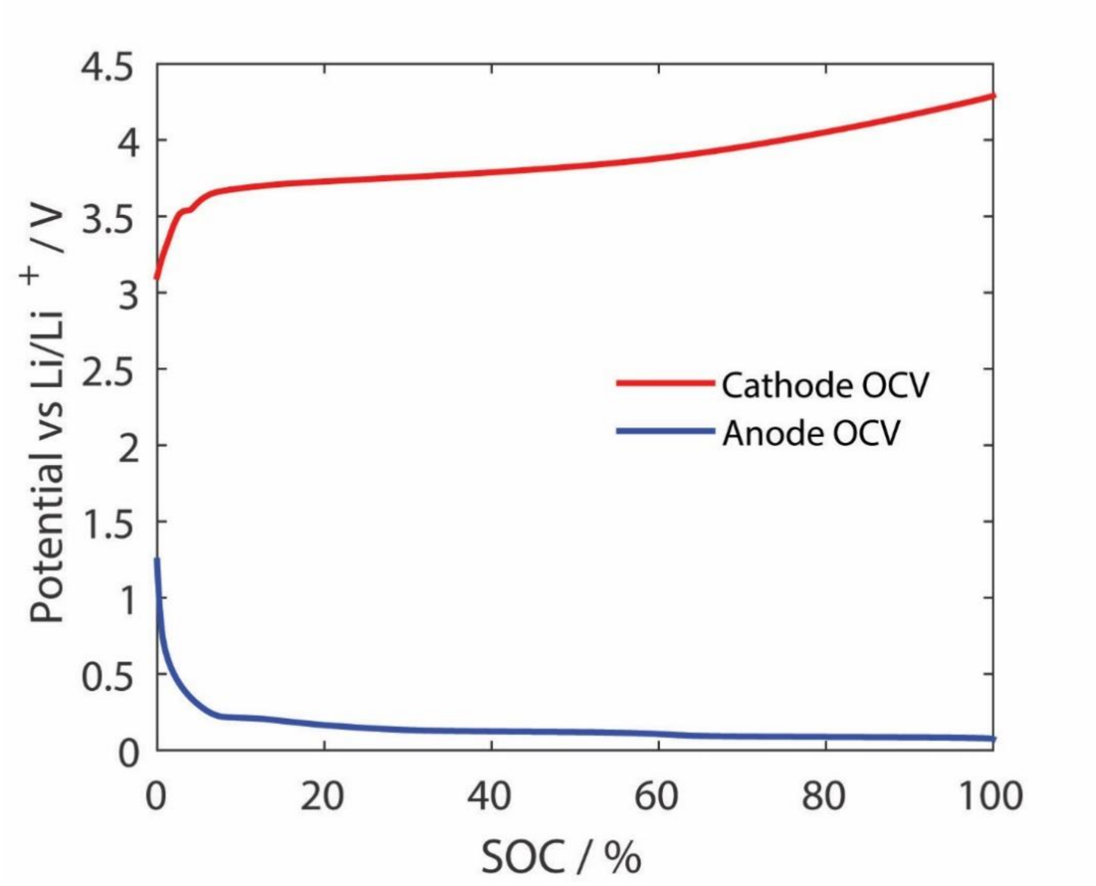

Supplementary Table S1. Summary of the experiments conducted on 16 different cells

No.	Terminal SOC	Terminal Voltage / V
1	0%	2.750
2	-10.7%	-2.185
3	-11.0%	-2.169
4	-12.1%	-2.108
5	-13.0%	-2.082
6	-13.7%	-2.026
7	-14.5%	-1.956
8	-14.8%	-1.930
9	-17.8%	-1.619
10	-20.0%	-1.198
11	-30.0%	-0.734
12	-40.0%	-0.611
13	-50.0%	-0.497
14	-60.0%	-0.476
15	-80.0%	-0.364
16	-100.0%	-0.358

Supplementary Table S2. Profile of the performance test

Step No.	Action	Duration	Condition	Cycle No.
1	Rest	10 min		
2	Constant current discharge		8.33 A(C/3) until 2.75 V	
3	Rest	60 min		
4	Constant current charge		8.33 A(C/3) until 4.2 V	
5	Constant voltage charge		4.2 V until $I < 0.5$ A	
6	Rest	60 min		
7	Constant current discharge		8.33 A(C/3) until 2.75 V	
8	Cycle		Steps 3-7	N
9	End			
